# Deliberate memory display can enhance conveyed value

**DOI:** 10.1111/bjop.12783

**Published:** 2025-02-27

**Authors:** Andrei I. Pintea, Devin G. Ray

**Affiliations:** ^1^ University of Aberdeen Aberdeen UK

**Keywords:** interpersonal interaction, interpersonal relationships, interpersonal value, memory, memory display

## Abstract

Letting someone else know that you value their presence, characteristics, effort or activities is central to building and maintaining human relationships. We investigated whether deliberate memory display is an effective means to convey such value. We examined these questions in the context of a simulated job interview (Experiments 1, 2 and 3, total *N* = 404) and a simulated ‘ice breaker’ exercise between new acquaintances (Experiment 4, total *N* = 156). Across experiments, results consistently indicated that memory display was not only an effective method of conveying value, but that memory display made other efforts to convey value more effective. Moreover, without external prompting, participants underutilized memory display despite its efficacy. These findings document the efficacy of memory display in the deliberate communication of value and suggest that deliberate memory display might be an underutilized strategic asset in the management of human relationships.

Mutual value is at the core of both personal and professional human relationships (Le & Agnew, 2003; Meyer & Allen, 1991). Letting someone else know that you value their presence, characteristics, effort or activities is thus a powerful and important message. Documented strategies for communicating value include flattery (Gordon, 1996), self‐disclosure (Collins & Miller, 1994), interest (Huang et al., 2017), responsiveness (Reis et al., 2004), support (Griffin & Hepburn, 2005) and sacrifice (Murray et al., 2006).

Memory display has also been linked to relational value. Sharing and discussing autobiographical memories creates and sustains closeness among friends and family (Bluck et al., 2019; Fivush, 2011). More directly, people appear to have a general belief that forgotten information is less valued than remembered information (Rhodes et al., 2017; Witherby et al., 2019). Moreover, people who feel they have been forgotten in some way feel less valued by and less close to the forgetter (Ray et al., 2019). Memory display thus appears to be an additional potential strategy that people might use to convey value. In the present work, we examine the efficacy and utilization of memory display as a strategy to convey value.

## THE VITAL IMPORTANCE OF CONVEYING VALUE

Feeling valued is an essential part of human relationships across both personal and professional contexts, and across casual, platonic, romantic and parental relationships. The importance of feeling valued is perhaps most clearly articulated in attachment theory and its derivatives. Attachment theory posits that accumulated signals of value from attachment figures lead to generalized beliefs about one's own value to other people (Bartholomew & Horowitz, 1991; Bowlby, 1973; Mikulincer & Shaver, 2007). A secure attachment style is formed when individuals are consistently made to feel worthy and deserving of love (i.e., valued) by their attachment figures. An insecure attachment style is formed by repeated interactions involving rejection, neglect or abuse from attachment figures (i.e., lack of value). Feeling either secure or insecure in one's value to others in turn predicts numerous dimensions of children's and adults' psychosocial functioning, including affective reactions, cognitive appraisals and relational behaviours (Mikulincer & Shaver, 2007; Sheinbaum et al., 2015).

New acquaintances commonly signal value by reciprocally disclosing personal information and experiences to one another, listening to each other's self‐disclosures, and demonstrating interest through follow‐up questions (Derlega et al., 2007; Huang et al., 2017; Sprecher et al., 2013; Sprecher & Treger, 2015). These behaviours convey value by indicating interest in and dedication to learning more about another person, and by demonstrating effort to establish common ground. Communicating value in these ways successfully predicts a range of affiliative outcomes, including mutual liking, closeness and perceived responsiveness (Collins & Miller, 1994; Huang et al., 2017; Sprecher et al., 2013; Sprecher & Treger, 2015), and increases the likelihood of future contact and relationship development (Altman & Taylor, 1973; Derlega et al., 2007). Conversely, work on ostracism and rejection makes clear that feeling devalued by both established and new acquaintances leads to a host of negative psychological and relational outcomes (see Williams, 2007 for a review).

The importance of conveying value in professional contexts has been studied less directly but is acknowledged in theories of organizational commitment (e.g., Meyer & Allen, 1991; Moreland & Levine, 2000). These theories posit that employees who feel valued and appreciated within their organization reciprocate with commitment to that organization. Similarly, leadership styles that acknowledge the importance of individual employee contributions are associated with job satisfaction and organizational commitment (Caldwell & Hayes, 2007; Haque et al., 2019; Lok & Crawford, 2004; Maxwell & Steele, 2003; Meyer et al., 2002).

It is thus no surprise that conveying value is an impactful action that tends to create virtuous cycles. Positive evaluations and expressions of liking are often reciprocated in kind (Byrne & Griffitt, 1973); reciprocal self‐disclosure between new acquaintances lays the foundation for relationship development (Derlega et al., 2007; Sprecher & Treger, 2015); expressions of gratitude increase relationship maintenance behaviours (Kubacka et al., 2011); and perceived reciprocity and support from an organization are better predictors of commitment than accumulated investments in an organization (Griffin & Hepburn, 2005). Conversely, conveying that another person is not valued damages relationships. Negative evaluations from close others predict later mental health problems (Vinokur & van Ryn, 1993) and even mere indifference threatens a variety of interpersonal and intrapersonal needs (Nezlek et al., 2012; Sheinbaum et al., 2015; Sommer et al., 2001).

## CONVEYING VALUE THROUGH MEMORY

Autobiographical memory is connected to the experience of value and closeness in interpersonal relationships. Shared memories and narratives form a key part of self‐definitions, and sharing memories and narratives creates closeness in new relationships and sustains closeness in existing relationships (Bluck et al., 2019; Fivush, 2011). Observing another person articulate memory for a shared experience is thus implicated in the communication of value.

More directly, memory has also been linked to explicit inferences of value. In non‐social contexts, people not only better remember more valuable information (Castel et al., 2009; Castel et al., 2011) but also consider information they remember to be more valuable than information they forget (Castel et al., 2012; Witherby et al., 2019). This ‘forgetting bias’ appears to be driven by a shared belief that forgotten information is less important than remembered information (Rhodes et al., 2017; Witherby et al., 2019).

The connection between memory and inferred value generalizes to interpersonal interaction. Ray et al. (2019) found that forgetting another person's characteristics, presence or past interactions conveyed that the forgotten information was relatively unimportant. The authors found that this effect was robust across (a) naturalistic investigation of forgetting in daily life, firsthand encounters in laboratory conditions and third‐party perceptions of forgetting, (b) relationships of different closeness, ranging from new acquaintances to family members and (c) different explanations for memory failure, including relational factors (e.g., lack of investment) and factors external to the relationship (e.g., forgetfulness, distraction). Observing evidence of another person's memory thus appears to be an influential signal of value in both social and non‐social contexts.

Despite the clear connection between perceived memory and conveyed value, however, we know of no investigation into the use of deliberate memory display for conveying value during interpersonal communication. We investigated this topic in the present work. Because memory effectively conveys value in interpersonal contexts, and because laypersons appear to be aware of this fact, we initially hypothesized that deliberate memory display would be an effective and frequently utilized means of conveying value.

As a related issue, we also examined the separability of links between memory display, conveyed value and liking. People tend to like others who remember their characteristics, presence and past interactions (Ray et al., 2019). Liking also plays a central role in other displays of value, for example, flattery (Gordon, 1996) and self‐disclosure (Huang et al., 2017; Sprecher & Treger, 2015). Conceptual analysis suggests that the link between memory display and liking is not perfect, however. For example, a display of memory for another person's transgressions is unlikely to make that person feel liked in the same way as a display of memory for more positive shared experiences. Additionally, liking encompasses many aspects of interpersonal interaction and value unrelated to memory (e.g., interpersonal warmth). Liking and memory display might thus be closely related or might operate with some degree of independence.

## DESIGN, STATISTICAL POWER AND ETHICS STATEMENT

All experiments had two phases. In the generation phase, participants (hereafter referred to as ‘generators’) generated a communication as part of a simulated interaction. Those communications were then coded for memory display. In the evaluation phase, new participants (hereafter referred to as ‘evaluators’) evaluated the content of the generators’ communications.

Pilot work suggested a large to very large effect size in the generation phase (*w* = 0.59). Sample sizes in the present individual experiments were sufficient to detect medium to large effects (*w* ≥ 0.44) with 80% power. Obtained single‐level effect sizes in the generation phase were large (Experiments 1–3, average *w* = 0.56) or medium‐large (Experiment 4, average *η*_*p*_^2^ = 0.10). Based on pilot work, recruitment was stopped once we were confident that the sample size would exceed 20 participants per between‐subjects condition.

We did not have a clear basis to estimate effect sizes in the evaluation phase. Sample size in the present individual experiments was thus sufficient to detect small effect sizes with 80% power (*f* ≥ .13), even assuming a conservative within‐participant response correlation of *r* = .20. Obtained single‐level effect sizes in the evaluation phase were large or very large (average *f* = .61). In Experiments 1 and 2, sample size was as large as possible given the resources available during the academic term in which data was collected. In Experiments 3 and 4, we kept sample size approximately consistent between experiments by stopping recruitment once we were confident that pre‐exclusion sample size would exceed 80 participants.

These experiments were approved by the authors' institutional ethics committee. The materials, data and scripts associated with this work, as well as with pilot work, are publicly accessible at https://osf.io/bjazd/?view_only=3b857b9f191749a4b3d6453e32137797. These studies were not pre‐registered.

## EXPERIMENT 1

Experiment 1 operationalized our questions in the context of a simulated unsuccessful job interview. We selected this context for several practical reasons. First, a job interview involves the review of extensive personal details in the form of qualifications and work history. This format thus provides substantial raw material with which to demonstrate memory. Second, the potential value placed on an unsuccessful candidate's time and effort is plausibly variable. Interviews can range from sham affairs where the successful candidate is predetermined to serious searches with reputational consequences for the hiring organization. Finally, the context is practically relevant. Interviews and feedback on interview performance are universally relevant to professionals and organizations.

### Method

#### Participants

Sixty‐three students from a university in the United Kingdom were recruited through university mailing lists and print advertisements to complete the generation phase of the experiment in exchange for £5. One of these generators did not ultimately provide a recorded communication. A second generator offered the job to the candidate (when they were instructed to do the reverse). These two cases were excluded from analyses, leaving a sample of 61 (41 women, 17 men, 3 unknown). Average age was 24.6 years, *SD* = 7.5, range: 17–68.

Eighty‐nine different students were recruited through social media and word of mouth to complete the evaluation phase of the experiment on a voluntary basis. Five of these evaluators were excluded from analyses for providing extensively incomplete responses, leaving a sample of 84 (61 women, 22 men, 1 unknown). Average age was 19.7 years, *SD* = 2.4, range:17–31.

#### Materials and procedure

In the generation phase, generators were asked to take the role of an employer looking for an office assistant and interviewing candidates for the position via video‐chat. They were told that they would watch a short (approx. 2 min) video recording of a candidate providing details about her qualifications and fit for the job (e.g., past work experience, relevant extracurricular activities from university). The video in question was created by asking an acquaintance of the first author to review her qualifications for a generic office job. Generators were additionally told that they would be asked to decide whether they wished to hire the candidate and to verbally communicate this decision to the candidate after watching the video. Generators then watched the video of the candidate reviewing her qualifications.

After the video, generators' instructions were changed. First, they were asked not to hire the candidate. Second, generators were given one of three additional prompts. Generators in a first condition (*convey value*) were asked to communicate to the candidate that the information provided by the candidate was important to them. Generators in a second condition (*display memory*) were asked to convey to the candidate that they remembered the candidate well through reference to specific details from the candidate's qualifications. Generators in a third condition (*disparage value*) were asked to convey to the candidate that the information provided by the candidate was not important to them.

We presented these amended instructions after generators had viewed the interview so that the instructions would not bias generators' perceptions of, or memory for, the interview. Following the amended instructions, generators were given 5 min with access to pen and paper to prepare (recorded) verbal feedback (approx. 1 min in length). These recordings were transcribed for coding.

Two naïve coders assessed the evidence of memory in each generator's feedback. The coders counted the number of references to details from the original stimulus interview that denoted unambiguous recall. Because the modal number of specific details referenced was zero, however, we ultimately opted to treat evidence of memory dichotomously. Agreement between the coders on the presence or absence of evidence of memory was acceptable (*κ* = .56, *p* < .001). For final analysis, discrepancies between the coders were resolved through discussion.

In the evaluation phase, evaluators were asked to rate the feedback provided by the generators. Evaluators were told only that generators (referred to as employers during the experiment) had provided feedback to an unsuccessful job candidate after seeing a recording of the candidate's interview. Evaluators then read a transcript of the stimulus interview, to which they could return whenever they wished. Evaluators then read and rated transcripts of the feedback provided by the generators. We relied on transcripts (as opposed to the original video) to preserve generator anonymity and to simplify the logistical demands of the experiment.

Evaluators were asked two questions about each transcript ‘How important was the interview to the employer?’, and, ‘How much did the employer like the interviewee?’ Both items used a seven‐point scale anchored at 1 (*not at all*) and 7 (*extremely*). The transcripts were evenly divided into two batches, with different experimental conditions represented as evenly as possible, and rated one at a time. In later experiments, with larger samples of generators, transcripts were instead divided into three batches.

### Results

Coder‐assessed memory was analysed using single‐level logistic regression (i.e., wide data). Evaluators' ratings were analysed using standard multilevel regression with random slopes for condition and random intercepts at the level of the subject (i.e., long data). Transcript batch was not included as a level in analysis of evaluator ratings because it did not show meaningful variance in this, or any later, experiments, Wald *Z*s <0.79, *p*s >0.434. Where estimates of variance for a particular condition's slope approached zero (and thus prevented accurate model convergence), the slope was instead treated as fixed.^1^

#### Coder assessment of memory

**FIGURE 1:**
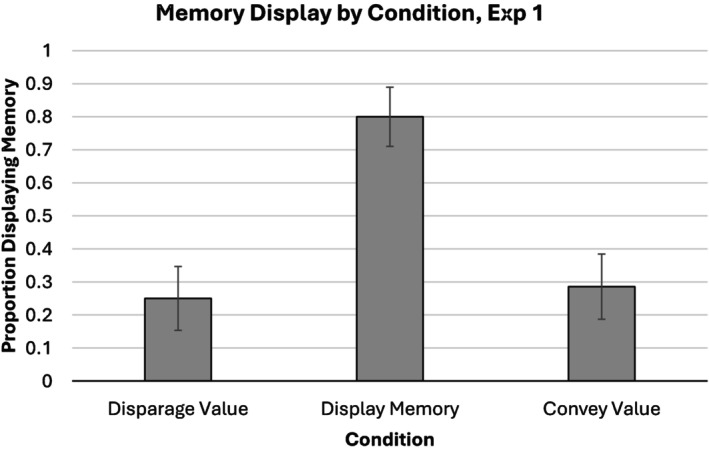
Proportion of participants displaying memory in the *Disparage Value* condition, the *Convey Value* condition and the *Display Memory* condition. Error bars represent standard errors. Most participants were capable of displaying memory when directly prompted, but only a minority of participants did so without prompting.

Based on our prediction that memory display would be frequently utilized, we expected more memory display in the ‘convey value’ and ‘display memory’ conditions than in the ‘disparage value’ condition. This prediction was contradicted (Figure 1). Condition had a clear effect on whether generators provided evidence of memory, *χ* ^2^(2, *N* = 61) = 16.12, Nagelkerke'*s R* ^2^ = 0.31, *p* < .001, but the ‘display memory’ condition (80%) showed more frequent memory than both the ‘disparage value’ condition (25%), *b*_logit_ = −2.49, Wald *Z* = 10.66, *p* = .001, OR = 0.08, 95% CI [0.02, 0.37], and the ‘convey value’ condition (29%), *b*_logit_ = −2.30, Wald *Z* = 9.71, *p* = .002, OR = 0.10, 95% CI [0.02, 0.42].

#### Evaluator rating of value

**FIGURE 2:**
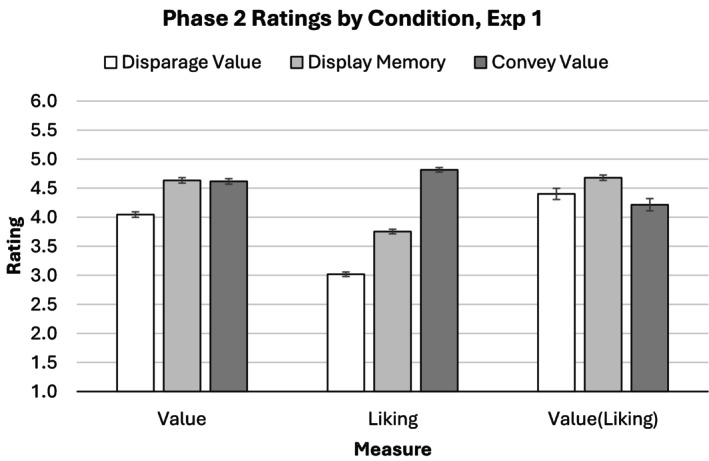
Phase 2 ratings of the value [*Value*], liking [*Liking*] and value adjusted for liking [*Value(Liking)*] conveyed by participants in Experiment 1. Error bars represent standard errors adjusted for within‐subjects variance according to the recommendations of O'Brien and Cousineau (2014). Participants prompted to display memory conveyed levels of value comparable to participants prompted to convey value. Additionally, controlling for the impact of apparent liking on conveyed value reduced the value conveyed by participants prompted to convey value but did not similarly impact the value conveyed by participants prompted to display memory.

Based on our prediction that memory display would be an effective means of conveying value, we expected value ratings in the ‘display memory’ condition to be higher than in the ‘disparage value’ condition. Evaluator ratings indicated that memory display was indeed an effective means of conveying value (Figure 2, left). The evaluators believed that generators in the ‘display memory’ condition ($\hat{M}$ = 4.64, 95% CI [4.40, 4.89]) conveyed more value than generators in the ‘disparage value’ condition, *b* = −0.54, 95% CI [−0.69, −0.40], *t*(90.79) = −7.60, *p* < .001. Moreover, the value conveyed by generators in the ‘display memory’ condition and the ‘convey value’ condition was rated comparably, *b* = −0.01, 95% CI [−0.16, 0.14], *t*(92.15) = −0.09, *p* = .926.

#### Evaluator ratings of liking

We did not make specific predictions about liking ratings. Evaluator ratings of liking responded more strongly to the ‘convey value’ instructions than to the ‘display memory’ instructions (Figure 2, centre). The evaluators believed that generators in the ‘display memory’ condition ($\hat{M}$ = 3.70, 95% CI [3.56, 3.83]) showed less liking for the interview candidate than generators in the ‘convey value’ condition, *b* = 1.20, 95% CI [1.06, 1.34], *t*(656.05) = 16.85, *p* < .001, but showed more liking for the interview candidate than generators in the ‘disparage value’ condition *b* = −0.68, 95% CI [−0.81, −0.54], *t*(119.61) = −9.92, *p* < .001.

#### Rated value controlling for rated liking

In order to better understand the relationship between evaluator ratings of value and liking, we reanalysed the effect of experimental condition on rated value controlling for rated liking (i.e., we predicted rated value from condition and liking simultaneously). This analysis suggested that displaying memory conveyed value independently of liking, at least in part. (Figure 2, right). After controlling for rated liking, the relative position of the ‘convey value’ condition fell. The adjusted value ratings indicated that the ‘display memory’ condition ($\hat{M}$ = 4.66, 95% CI [4.43, 4.90]) conveyed more value than the ‘convey value’ condition, *b* = −0.29, 95% CI [−0.41, −0.18], *t*(101.75) = −5.15, *p* < .001. The relative standing of the ‘display memory’ condition and the ‘disparage value’ condition remained unchanged, however, *b* = −0.40, 95% CI [−0.52, −0.29], *t*(100.41) = −6.82, *p* < .001.

### Discussion

We hypothesized that deliberate memory display would be an effective means of conveying value. This hypothesis was supported. When participants took the role of an employer providing feedback to an unsuccessful interview candidate, the value conveyed by their feedback was rated comparably regardless of whether they had been instructed to demonstrate memory for the candidate's interview or to let the candidate know her interview had been valued.

We also hypothesized that deliberate memory display would be a frequently utilized method of conveying value. Surprisingly, this hypothesis was not supported. Most participants who were instructed to let the candidate know her interview had been valued did not utilize memory display in their communication. In fact, these participants displayed memory no more frequently than did participants instructed to let the candidate know that her interview had *not* been valued. Importantly, participants were capable of demonstrating memory if they chose to; the large majority of participants who were directly instructed to demonstrate memory as part of their communication did so.

The results of Experiment 1 also suggest the intriguing possibility that memory display and liking might be non‐redundant methods of conveying value. Liking ratings responded more strongly to instructions asking participants to let the candidate know her interview had been valued than to instructions asking participants to demonstrate that they remembered details of the candidate's interview. Moreover, controlling for rated liking reduced the impact of instruction to let the candidate know she was valued on value ratings, but did not similarly reduce the impact of instructions to demonstrate memory for the interview. In combination with the finding that participants underutilized memory display when instructed to let the candidate know they valued her interview, these findings suggest that memory display might be combined with participants' default liking‐based approach in order to convey greater value than either displaying memory or showing liking would convey alone.

## EXPERIMENT 2

Experiment 2 focused on the possibility that memory display and liking‐based strategies might be non‐redundant methods of conveying value. If memory display were an underutilized strategy that did not rely on liking, then it might be possible to use memory display to make liking‐based strategies more effective at conveying value. Based on this reasoning, and on the results of Experiment 1, we predicted that the combination of memory display with the ‘convey value’ instructions from Experiment 1 would lead to more memory display and more conveyed value than the ‘convey value’ instructions alone.

### Method

#### Participants

Fifty‐four students from a university in the United Kingdom participated in the generation phase of the experiment in exchange for £5. One generator did not ultimately provide a recorded communication, and the recordings of two more generators were discarded due to technical issues during testing. The final sample consisted of 51 generators (13 men, 37 women, 1 non‐binary designation). Participants were 22.8 years old on average (*SD* = 6.8, range: 17–64).

Sixty‐three people participated in the evaluation phase of the experiment in exchange for £2.70. Participants were recruited from Prolific Academic (https://prolific.co), a crowdsourcing website for conducting online research. Five evaluators were excluded from the analysis for the following reasons: providing extensive incomplete responses (*n* = 2), abnormally short study completion times (<5 min; *n* = 1), or duplicate submissions (*n* = 2). The final sample consisted of 58 evaluators (26 women, 32 men). Participants were 35.0 years old on average (*SD* = 12.2, range: 18–74).

#### Materials and procedure

Experiment 2 compared a condition identical to the ‘convey value’ condition in Experiment 1 to a new *convey value using memory display* condition. In the ‘convey value using memory display’ condition, generators were instructed to communicate to the candidate that the information provided by the candidate was important to them *by* indicating that they remembered the candidate well through reference to specific details of the candidate's qualifications. The materials and procedure in these conditions were otherwise identical to Experiment 1.

Two naïve coders evaluated the evidence of memory present in each generator's feedback. As in Experiment 1, we treated evidence of memory dichotomously. Agreement between the coders on the presence or absence of evidence of memory was acceptable (*κ* = .61, *p* < .001). For final analysis, discrepancies between the coders were resolved through discussion.

### Results

#### Coder assessment of memory

Based on the results of Experiment 1, we expected relatively infrequent memory display in the ‘convey value’ condition and relatively frequent memory display in the ‘convey value using memory’ condition. This prediction was supported, χ^2^(1, *N* = 51) = 13.62, Nagelkerke's *R* ^2^ = 0.33, *p* < .001. Fewer than half of generators in the ‘convey value’ condition (46%) displayed evidence of memory but the large majority of generators in the ‘convey value using memory’ condition (92%) displayed evidence of memory, *b*_logit_ = −2.60, Wald *Z* = 9.67, *p* = .002, *OR* = 0.08, 95% CI [0.01, 0.38].

#### Evaluator ratings of value and liking

Because memory display was underutilized without prompting, we expected that the addition of memory display to the ‘convey value’ instruction would enhance the effectiveness of the ‘conveyed value’ instructions. As predicted, the evaluators believed that generators in the ‘convey value using memory’ condition ($\hat{M}$ = 4.26, 95% CI [3.94, 4.57]) conveyed more value than generators in the ‘convey value’ condition *b* = −0.17, 95% CI [−0.29, 0.04], *t*(52.83) = −2.72, *p* = .009. Evaluator did not believe, however, that participants in the ‘convey value using memory’ condition ($\hat{M}$ = 4.18, 95% CI [3.95, 4.41]) showed detectably more liking than participants in the ‘convey value’ condition, *b* = −0.05, 95% CI [−0.19, 0.08], *t*(1310.07) = −0.81, *p* = .421. Moreover, the difference in value ratings between the two conditions held when controlling for liking ratings, *b* = −0.18, 95% CI [−0.29, −0.07], *t*(54.80) = −3.17, *p* = .003.

### Discussion

The present results corroborate and extend Experiment 1. Coder assessment of displayed memory and evaluator ratings of value together indicated that memory display was not only an effective but underutilized strategy for conveying value, but that memory display could enhance the effectiveness of other efforts to convey value. Moreover, evaluator ratings of liking indicated that the added benefit of memory display could not be accounted for by liking.

## EXPERIMENT 3

Experiment 3 replicated and extended Experiments 1 and 2 using a more naturalistic pairing between experimental prompts and context. In previous experiments, generators were prompted to convey value without a clear purpose or explanation as to how their actions were relevant to their task as an interviewer. The decontextualized nature of these prompts might have deprived generators of contextual cues that would spur them to utilize memory display more spontaneously. Additionally, Experiment 3 included all four of the experimental conditions used across Experiments 1 and 2 in a single design.

### Method

#### Participants

Eighty‐three students from a university in the United Kingdom were recruited through a departmental participant pool to complete the generation phase in exchange for partial course credit. One generator was excluded because they did not ultimately provide a response, leaving a sample of 82 (11 men, 70 women, 1 undisclosed). Participants had a mean age of 19.1 (*SD* = 1.3, range: 17–23).

Eighty‐one participants recruited from Prolific Academic participated in the evaluation phase in exchange for £1.75. Thirteen evaluators were excluded from the analyses for the following reasons: providing extensive incomplete submissions (*n* = 2), abnormally short study completion durations (<5 min; *n* = 1), or failing an attention check (*n* = 10). The final sample consisted of 68 evaluators (28 men, 40 women). Evaluators had a mean age of 32.3 (*SD* = 12.5, range: 16–63).

#### Materials and procedure

Experiment 3 was identical to Experiments 1 and 2, with the following exceptions. First, instructions prompting generators to convey value were contextualized. Generators in the ‘convey value’ condition were told that company policy considered interviews to be part of public relations, and even unsuccessful candidates should thus be made to feel valued and appreciated. In the ‘convey value using memory’ condition, Generators were additionally told that an effective strategy to make candidates feel valued and appreciated was to make clear that they remembered the candidate's interview well by reference to specific details of the candidate's qualifications. Second, instead of a ‘disparage value’ condition, Experiment 3 contained a control condition in which generators were told only to provide feedback to the interview candidate with no reference to conveying value or displaying memory. Third, for ease of administration, generators read a transcript of a candidate reviewing her qualifications for an office assistant position rather than viewing a video and generators prepared written (rather than verbal) feedback. Fourth, phase 2 ratings contained an attention check. The attention check consisted of text that initially appeared to be a generator response presented for evaluation, but continued by instructing participants to select ‘prefer not to say’ for both liking and value ratings.

As in the previous experiments, two naïve coders evaluated the presence or absence of evidence of memory in each original participant's feedback. Agreement between the coders on the presence or absence of evidence of memory was high (*κ* = .80, *p* < .001). For final analysis, discrepancies between the coders were resolved through discussion.

### Results

#### Coder assessment of memory

As in previous experiments, generators did not reliably utilize memory display unless directly instructed to do so, *χ* ^2^(1, *N* = 82) = 19.142, Nagelkerke's *R* ^2^ = 0.30, *p* < .001. Memory display occurred less often in the ‘convey value’ condition (55%), *b*_logit_ = −2.10, Wald *Z* = 5.88, *p* = .015, OR = 0.12, 95% CI [0.02, 0.67], and in the control condition (42%), *b*_logit_ = −2.62, Wald *Z* = 8.97, *p* = .003, OR = 0.07, 95% CI [0.01, 0.40], than in the ‘display memory’ condition (91%). Participants displayed comparably high levels of memory in the ‘display memory’ and ‘convey value using memory display’ (90%) conditions, however, *b*_logit_ = −0.51, Wald *Z* = 0.00, *p* = .961, OR = 0.95, 95% CI [0.12, 7.44].

#### Evaluator ratings of value

**FIGURE 3:**
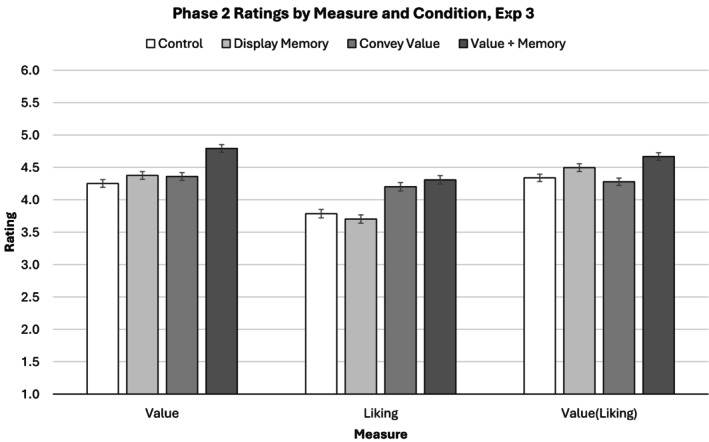
Phase 2 ratings of the value [*Value*], liking [*Liking*] and value adjusted for liking [*Value(Liking)*] conveyed by participants in Experiment 3. Error bars represent standard errors adjusted for within‐subjects variance according to the recommendations of O'Brien and Cousineau (2014). Participants prompted to display memory and participants prompted to convey value conveyed comparable levels of value, whereas participants prompted to convey value through memory display conveyed more value than all other conditions. Liking ratings and reanalysis of value ratings controlling for liking suggest that memory display did not influence value through liking, although liking was an effective strategy to convey value.

Evaluator ratings of value again responded similarly to the ‘display memory’ and ‘convey value’ instructions (Figure 3, left). Evaluators believed that generators in the ‘display memory’ condition and generators in the ‘convey value’ condition conveyed comparable levels of value, *b* = −0.03, 95% CIs [−0.18, 0.13], *t*(90.46) = −0.37, *p* = .709. Additionally, rated value again responded most strongly to the ‘convey value using memory display’ condition. Evaluators believed that generators in the ‘convey value using memory’ condition ($\hat{M}$ = 4.84, 95% CI [4.57, 5.12]) conveyed more value than generators in both the ‘display memory’ condition, *b* = −0.38, 95% CIs [−0.54, −0.23], *t*(92.41) = −4.88, *p* < .001, and the ‘convey value’ condition, *b* = −0.42, 95% CIs [−0.59, −0.26], *t*(91.98) = −5.05, *p* < .001.

Experiment 3 differed from Experiment 1 in that the rated value in the control condition did not definitively differ from the ‘display memory’ condition, *b* = −0.09, 95% CIs [−0.27, 0.10], *t*(79.00) = −0.90, *p* = .372. Critically, the control condition in Experiment 3 also differed from Experiment 1. The Experiment 1 control condition instructed participants to actively avoid conveying value, whereas the Experiment 3 control condition gave no instructions about conveying value or displaying memory.

#### Evaluator ratings of liking

Evaluator ratings of liking responded more strongly to the ‘convey value’ and ‘convey value using memory display’ instructions than to the ‘display memory’ instructions (Figure 3, middle). Evaluators believed that generators in the ‘display memory’ condition ($\hat{M}$ = 3.86, 95% CI [3.65, 4.06]) liked their interaction partner less than generators in both the ‘convey value’ condition, *b =* 0.22, 95% CI [0.06, 0.38], *t* = 2.74, *p* = .006, and the ‘convey value using memory display’ condition, *b* = 0.31, 95% CIs [0.10, 0.52], *t* = 3.05, *p* = .003. Liking ratings in the ‘display memory’ condition were not detectably different from the control condition, *b* = −0.04, 95% CIs [−0.21, 0.14], *t*(735.17) = −0.427, *p* = .669 and liking ratings in the ‘convey value’ condition ($\hat{M}$ = 4.08, 95% CI [3.88, 4.28]) were not detectably different from the ‘convey value using memory’ condition, *b* = 0.09, 95% CIs [−0.12, 0.29], *t*(98.06) = 0.816, *p* = .417.

#### Value ratings controlling for liking ratings

Reanalysis of the effect of experimental condition on value ratings controlling for liking ratings reduced the impact of the ‘convey value’ and ‘convey value using memory display’ conditions on rated value relative to the ‘memory display condition’, although not as dramatically as in previous experiments (Figure 3, *right*). Although reduced in magnitude, the difference in value ratings between the ‘display memory’ condition ($\hat{M}$ = 4.47, 95% CI [4.23, 4.70]) and the ‘convey value using memory display’ condition remained significant, *b* = 0.21, 95% CIs [0.05, 0.37], *t*(74.51) = 2.65, *p* = .009. Similarly, although the position of the ‘convey value’ condition fell relative to the ‘display memory’ condition, the emerging difference in value ratings between these conditions did not reach traditional thresholds of statistical significance, *b* = −0.12, 95% CIs [−0.26, 0.02], *t*(83.35) = −1.76, *p* = .083.

### Discussion

Experiment 3 replicated all five key findings from Experiments 1 and 2 using less artificial manipulations. First, deliberate memory display was again an effective means of conveying value; evaluator ratings of value indicated that generators instructed to display memory conveyed value just as effectively as generators directly instructed to make the interview candidate feel valued. Second, memory display was again underutilized without explicit instruction; memory display was unreliable among generators instructed only to make the candidate feel valued but nearly universal among generators directly instructed to display memory. Third, efforts to make the interview candidate feel valued without memory display involved liking; instruction to make the candidate feel valued elevated liking ratings relative to instruction to display memory, and the impact of these instructions on value ratings was accounted for by liking ratings. Fourth, memory display was not closely related to liking; instruction to display memory did not elevate liking ratings relative to other conditions, and liking did not account for the effects of these instructions on value ratings. Fifth and finally, memory display enhanced the effectiveness of liking‐based efforts to convey value; generators instructed to make the candidate feel valued by displaying memory conveyed more value than generators instructed to convey value without reference to memory display.

Although findings from Experiments 1–3 are consistent and intriguing, all three of these experiments relate to a very specific context – providing feedback to an unsuccessful job candidate. This context is professional, formal and requires rejection. Interpersonal liking and memory display might be less distinct, and people might more naturally draw on memory display to convey value in more personal, informal and positive contexts.

## EXPERIMENT 4

Experiment 4 adapted our paradigm to the context of getting to know a new acquaintance. This type of interaction is personal rather than professional, casual rather than formal and requires no element of rejection. Experiment 4 also added evaluator‐rated memory to the evaluation phase so that we could compare memory and liking on the same metric. Experiment 4 retained all conditions present in Experiment 3.

### Method

#### Participants

Eighty‐seven students (12 men, 73 women, 2 unknown) from a university in the United Kingdom participated in the generation phase in exchange for partial course credit. These generators had a mean age of 19.46 (*SD* = 2.25, range: 18–35).

Eighty‐seven additional participants recruited from Prolific Academic participated in the evaluation phase in exchange for £2.20. Nineteen evaluators were excluded from the analyses for the following reasons: abnormally short study completion durations (<7 min; *n* = 5), duplicate submissions (*n* = 1) and failing an attention check (*n* = 13). The final sample consisted of 68 participants (36 men, 32 women). These evaluators had a mean age of 27.6 years (*SD* = 10.1, range: 18–64).

#### Materials and procedure

Experiment 4 followed the same two‐phase structure as previous experiments. In the generation phase, participants' task was to get acquainted with a new acquaintance through repeated exchange of personal information. The interaction consisted of six increasingly intimate question‐and‐answer exchanges taken from the Closeness‐Generating Procedure (Aron et al., 1997) and the Relationship Closeness Induction Task (Sedikides et al., 1999). The interaction partner was simulated through scripted responses, which were based on participants' responses in a pilot study. Generators in the present study were told that the interaction was simulated but were asked to treat it as though it were real. After the interaction was over, generators were asked to provide a final response in which they communicated their thoughts about the interaction and about the interaction partner to their interaction partner.

Generators were then given 5 min to prepare a written response and received additional instructions about conveying value and memory display. The instructions were the same as those used in Experiment 3, except that the ‘convey value’ instructions asked only that generators make their interaction partner feel valued and appreciated.

Two coders who were blind to condition evaluated the evidence of memory present in each generator's communication. In contrast to previous experiments, almost all generators in Experiment 4 made at least one reference to specific details from the interaction. Consequently, evidence of memory was treated continuously by counting the number of unique details to which participants referred. Agreement between coders on memory count was high (*κ* = .71, *p* < .001). Discrepancies between the coders were resolved through discussion.

In the evaluation phase, evaluators rated transcripts of generators' responses. In addition to rating conveyed value and liking, evaluators were also asked, ‘How well did the participant [generator] remember their interaction partner's answers?’. Like the other ratings, this item used a seven‐point scale anchored at 1 (*not at all*) and 7 (*extremely*).

### Results

#### Coder assessment of memory

Across all conditions, memory display was much more frequent than in previous experiments (*M* = 3.69, *SD* = 2.20). As in previous experiments, however, generators underutilized their capacity for memory display unless they were specifically instructed to display memory. A one‐way between‐subjects ANOVA with four levels (display memory, convey value, convey value using memory and control) on the frequency of coder‐assessed memory display yielded an effect of condition, *F*(3, 83) = 13.18, *p* < .001, *ηp* ^2^ = .32, 90% CI [.17, .42]. Memory display in the ‘display memory’ condition (*M* = 4.88, *SD* = 1.83) was comparable to the ‘convey value using memory’ condition (*M* = 4.62, *SD* = 1.67), *t*(45.61) = 0.50, *p* = .944, *d* = 0.14, 95% CI [−0.42, 0.71], but was greater than memory display in the ‘convey value’ condition (*M* = 3.00, *SD* = 2.17), *t*(32.96) = 2.96, *p* = .003, *d* = 0.95, 95% CI[0.28, 1.59] and in the control condition (*M* = 1.86, *SD* = 1.74), *t*(42.66) = 5.65, *p* < .001, *d* = 1.69, 95% CI [1.00, 2.37]. The ‘convey value’ condition also tended to display more memory than the control condition, although this difference did not reach conventional levels of statistical significance, *t*(32.52) = 1.79, *p* = .082, *d* = .0.14, 95% CI [−0.06, 1.23].

#### Evaluator ratings of memory

**FIGURE 4:**
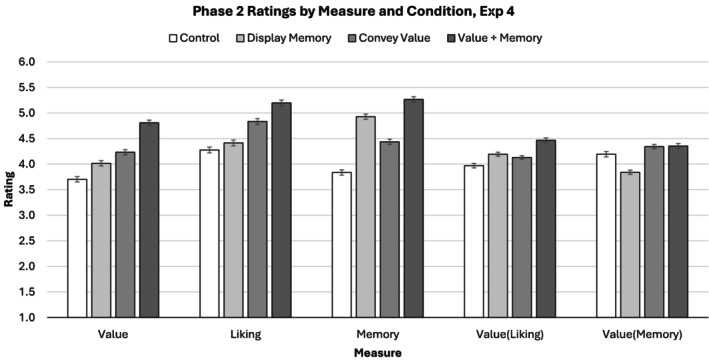
Phase 2 ratings of the value [*Value*], liking [*Liking*] and memory [*Memory*], as well as of value adjusted for liking [*Value(Liking)*]and value adjusted for memory [*Value(Memory)*], conveyed by phase 1 participants in Experiment 4. Error bars represent standard errors adjusted for within‐subjects variance according to the recommendations of O'Brien and Cousineau (2014). Participants prompted to display memory conveyed more value than participants in the control condition, and participants prompted to convey value through memory display conveyed more value than all other conditions. Liking ratings, reanalysis of value controlling for liking, and reanalysis of value controlling for memory suggested that memory display influenced value directly through apparent memory rather than through liking, although liking remained an effective strategy to communicate value.

Evaluator ratings of memory mirrored coder‐assessed memory in most ways but suggested a larger impact of instruction to convey value (Figure 4, middle). The evaluators believed that generators in the ‘display memory’ condition ($\hat{M}$ = 5.00, 95% CI [4.80, 5.21]) displayed more memory than generators in the ‘convey value’ condition, *b* = −0.45, 95% CIs [−0.63, −0.27], *t*(617.37) = −4.91, *p* < .001 and in the control condition, *b* = −1.05, 95% CIs [−1.24, 0.87], *t*(730.61) = −11.10, *p* < .001, but less memory than generators in the ‘convey value using memory display condition’, *b* = 0.29, 95% CIs [0.14, 0.44], *t*(128.60) = 3.75, *p* < .001. Additionally, the evaluators believed that generators in the ‘convey value’ condition ($\hat{M}$ = 4.55, 95% CI [4.33, 4.77]) displayed more memory than generators in the control condition, *b* = −0.60, 95% CIs [−0.81, −0.40], *t*(644.58) = 5.74, *p* < .001.

#### Evaluator ratings of value

Evaluator ratings of value mirrored previous experiments in most ways but suggested a larger relative impact of instructions to convey value (Figure 4, left). Evaluators believed that generators in the ‘memory display’ condition ($\hat{M}$ = 4.01, 95% CI [3.76, 4.27]) conveyed more value than generators in the control condition, *b* = −0.20, 95% CIs [−0.37, −0.03], *t*(761.53) = −2.37, *p* = .018, but less value than generators in the ‘convey value’ condition, *b* = 0.19, 95% CIs [0.02, 0.35], *t*(797.61) = 2.21, *p* = .028. As in previous experiments, however, evaluators believed that generators in the ‘convey value using memory display’ condition ($\hat{M}$ = 4.74, 95% CI [4.49, 4.99]) conveyed more value than generators in both the ‘display memory’ condition, *b* = −0.72, 95% CIs [−0.87, −0.57], *t*(933.95) = −9.45, *p* < .001, and in the ‘convey value’ condition, *b* = −0.54, 95% CIs [−0.70, −0.38], *t*(765.19) = −6.50, *p* < .001.

#### Evaluator ratings of liking

Evaluator ratings of liking mirrored previous experiments in most ways (Figure 4, middle‐left). Liking ratings were comparable in the ‘memory display’ condition ($\hat{M}$ = 4.46, 95% CI [4.24, 4.68]) and control condition, *b* = −0.10, 95% CIs [−0.26, 0.06], *t*(752.87) = −1.26, *p* = .208, and liking ratings in the ‘display memory’ condition were lower than in the ‘convey value condition’, *b* = 0.31, 95% CIs [0.14, 0.47], *t*(113.07) = 3.57, *p* < .001. At the same time, however, rated liking in Experiment 4 appeared to be more influenced by memory display than in previous experiments. Rated liking was higher in the ‘convey value using memory display’ condition ($\hat{M}$ = 5.18, 95% CI [4.98, 5.38]) than in the ‘convey value’ condition, *b* = −0.42, 95% CIs [−0.58, −0.25], *t*(95.43) = −5.00, *p* < .001.

#### Rated value controlling for rated liking

As in previous experiments, reanalysis of rated value controlling for rated liking suggested that rated liking and rated value showed more overlap in the ‘convey value’ and ‘convey value using memory display’ conditions than in the ‘display memory’ condition (Figure 4, middle‐right). The previous difference in conveyed value between the ‘display memory’ condition ($\hat{M}$ = 4.20, 95% CI [4.03, 4.36]) and the ‘convey value’ condition was reduced to non‐significance, *b* = −0.03, 95% CIs [−0.14, 0.07], *t*(750.73) = −0.66, *p* = .507. Similarly, the previous difference in conveyed value between the ‘convey value using memory display’ condition ($\hat{M}$ = 4.32, 95% CI [4.16, 4.49]) and the ‘display memory’ condition was substantially reduced, although it remained significant, *b* = −0.13, 95% CIs [−0.22, −0.03], *t*(986.88) = −2.68, *p* = .008.

#### Rated value controlling for rated memory

Reanalysis of the effect of experimental condition on rated value controlling for rated memory suggested more overlap between rated memory and rated value in the ‘display memory’ and ‘convey value using memory’ conditions than in the ‘convey value’ and control condition (Figure 4, right). The previous difference in rated value between the ‘convey value using memory display’ condition ($\hat{M}$ = 4.43, 95% CI [4.23, 4.64]) and the ‘convey value’ condition was reduced to non‐significance, *b* = 0.00, 95% CIs [−0.12, 0.12], *t*(706.46) = 0.05, *p* = .959. Conversely, the previous difference between the ‘display memory’ condition ($\hat{M}$ = 3.94, 95% CI [3.73, 4.15]) and the ‘convey value’ condition was exaggerated, *b* = 0.49, 95% CIs [0.37, 0.62], *t*(728.39) = 7.70, *p* < .001.

### Discussion

These results replicated all five key findings from previous experiments in a personal and informal context that did not require an element of rejection. First, memory display remained an effective means of conveying value; although generators instructed to display memory were rated as conveying less value than generators instructed to make their interaction partner feel valued, generators instructed to display memory were rated as conveying more value than generators in the control condition. Second, memory display was underutilized without instruction; although overall frequency was higher than in previous experiments, both coder assessment and evaluator ratings indicated that generators who were instructed to display memory still displayed more memory than generators instructed only to make their interaction partner feel valued. Third, generators' efforts to make their interaction partner feel valued involved liking; generators focused solely on making their partner feel valued were rated as showing more liking than generators focused solely on memory display, and controlling for the effects of liking on value ratings reduced the relative effectiveness of instructions focused on conveying value more than instructions focused on memory display. Fourth, memory display was at least partially independent of liking; controlling for liking ratings did not fully account for the effect of memory display on value ratings. Fifth, the combination of memory display and liking‐based strategies conveyed greater value than either strategy alone; generators instructed to convey value using memory display were rated as conveying more value than generators instructed to convey value without reference to memory display, and more value than generators instructed to display memory without reference to conveying value.

Experiment 4 also suggests that these key findings were sensitive to context. In the more personal, informal and positive context of Experiment 4, there appeared to be more overlap between memory display and liking than in previous experiments. Not only did the ‘memory display’ instructions elevate liking ratings, but the ‘convey value’ instructions elevated memory ratings. Similarly, Experiment 4's generally elevated levels of memory display suggested that memory display came more naturally in a personal, informal and positive interaction than in a professional and formal communication about rejection.

## GENERAL DISCUSSION

In four experiments, we investigated whether deliberate memory display would effectively convey value during interpersonal communication. We hypothesized that deliberate memory display would be an effective and frequently utilized means of conveying value. We also explored the separability of links between memory, conveyed value and liking. Experiments 1 through 3 examined these questions in the context of rejecting an unsuccessful job interview candidate. Experiment 4 generalized our investigation to the context of getting to know a new acquaintance.

Memory display, by itself, conveyed value effectively. In the context of providing feedback to an unsuccessful interview candidate, instruction to display memory and instruction to let the candidate know that her time had been valued led to comparable value ratings and higher value ratings than instruction to devalue the interview. In the context of getting to know a new acquaintance, instruction to display memory led to higher value ratings than no instruction.

The frequency with which memory display was spontaneously deployed during efforts to convey value was more variable than we expected. Participants did not reliably use memory display when they were trying to show an unsuccessful interview candidate that her time had been valued. Importantly, this was not because these participants lacked capability; participants directly instructed to display memory in this context were reliably able to do so. In contrast, when getting to know a new acquaintance, memory display was common across all instruction sets. Moreover, participants showed more memory when trying to convey value to an acquaintance than in the control condition. Importantly, however, participants directly instructed to display memory displayed even higher levels of memory. Taken together, these results suggest (a) that the spontaneous use of memory display during efforts to convey value is by no means universal and (b) that even in contexts where memory display is common, people will not always draw on their full capacity for memory display when trying to convey value.

Our findings also suggest a reason for why participants did not spontaneously utilize their full capacity for memory display—participants instead focused on being nice. Across contexts, participants' unguided efforts to convey value involved making it clear that they liked their interaction partner much more reliably than they involved memory display. Importantly, the addition of memory display did not undermine this default strategy in any way. Rather, with minimal guidance, participants were capable of both conveying liking and displaying memory at the same time.

Notably, this synergy allowed participants to convey value more effectively. In all experiments, instruction to convey value using memory display conveyed more value than instruction to convey value without reference to memory display.

In sum, people who were trying to make someone else feel valued as part of communication usually did not mention as many details about a previous shared experience as they could have. When people were encouraged to include more of such details in their communication, they were able to do so and more effectively conveyed that they valued the communication recipient above and beyond indications of personal liking.

### Limitations and future directions

Our paradigms relied on simulated social interaction. The relevance of such simulation to real‐world behaviour is apparent in the wide use of simulation to teach effective technical and social skills (e.g., Gebhard et al., 2018; Turkelson et al., 2020). Simulations are, however, not the same thing as real‐world behaviour. A clear next step is thus to replicate the present findings in dyadic interactions between two participants.

These experiments relied on Western European samples, and the generation phase of these experiments relied exclusively on samples of university students. These samples carry with them attendant concerns about generalizability across cultures and across contexts, especially as undergraduate students are unlikely to have had real‐world experience with the hiring scenario used in Experiments 1–3.

We observed memory display more often in the relational context of getting to know a new acquaintance than in the task‐focused context of providing feedback to an unsuccessful interview candidate, although deliberate memory display helped participants to convey that they valued their interaction partner in both contexts. The greater frequency of memory display in a relational context might reflect the important role that explicit discussion of autobiographical memory plays in interpersonal relationships (Bluck et al., 2019; Fivush, 2011). An interesting additional test of the generalizability of the present effects would thus be to examine coached memory display during explicit discussion of a shared past in the context of already established relationships. If people were to underutilize memory display in these circumstances, it would suggest that the presently observed effects are robust even in circumstances that maximally support spontaneous memory display.

We explored a distinction between liking and conveyed value that is, to our knowledge, a novel one. The specific nature of this distinction cannot be defined precisely in the present work because we relied entirely on evaluator ratings to define liking and because evaluators were not asked to justify their ratings. One possibility is that this specific distinction might reflect a general distinction between behavioural demonstrations of investment (i.e., showing that you bothered to listen and remember) and tone of communication (i.e., interpersonal warmth). This explanation is, however, speculative and requires examination in future work.

We have defined memory display operationally as the verbal mention of specific details from past interactions. There is substantial room for theoretical elaboration on this definition. Are some details more effective to highlight than others? Does the duration of storage matter? Is the perception of memory linear such that more details always lead to greater perceived memory, or does the inclusion of additional detail yield diminishing returns? These questions, and others like them, raise exciting possibilities for future work.

The present results also raise exciting possibilities for application. It is possible that encouraging memory display in daily life might help people to more effectively make their colleagues, friends and loved ones feel valued. This would be a powerful intervention. New acquaintances who communicate value are liked more and viewed as closer (Sprecher & Treger, 2015). Romantic partners who better communicate value seem more responsive (Gordon et al., 2012; Murray et al., 2006) and more committed to their relationship (Le & Agnew, 2003). Organizations and managers who appear to value employees have better retention and more positive office dynamics (Eisenberger et al., 2001; Settoon et al., 1996). Even students who feel valued and supported by their teachers show increased learning and more positive teacher evaluations (Frymier & Houser, 2000; Teven & McCroskey, 1997). If confirmed in other paradigms, the present results thus suggest great potential for meaningful application.

## AUTHOR CONTRIBUTIONS

**Andrei I. Pintea:** Investigation; formal analysis; project administration; writing – original draft; methodology; data curation. **Devin G. Ray:** Conceptualization; methodology; formal analysis; writing – review and editing; funding acquisition.

## Data Availability

The data that support the findings of this study are openly available in Open Science Foundation at https://osf.io/bjazd/?view_only=3b857b9f191749a4b3d6453e32137797.

## Footnotes

^1^
This approach is the reason that degrees of freedom may appear discrepant between analyses.
